# Histopathological Findings in Immunohistological Staining of the Granulomatous Tissue Reaction Associated with Tuberculosis

**DOI:** 10.1155/2014/858396

**Published:** 2014-01-05

**Authors:** Shirin Karimi, Masoud Shamaei, Mihan Pourabdollah, Makan Sadr, Mehrdad Karbasi, Arda Kiani, Moslem Bahadori

**Affiliations:** ^1^Mycobacteriology Research Center, National Research Institute of Tuberculosis and Lung Diseases (NRITLD), Shahid Beheshti University of Medical Sciences, Tehran, Iran; ^2^Clinical Tuberculosis and Epidemiology Research Center, NRITLD, Shahid Beheshti University of Medical Sciences, Tehran, Iran; ^3^Pediatric Respiratory Diseases Research Center, National Research Institute of Tuberculosis and Lung Diseases (NRITLD), Shahid Beheshti University of Medical Sciences, Tehran, Iran; ^4^Tracheal Diseases Research Center, National Research Institute of Tuberculosis and Lung Diseases (NRITLD), Shahid Beheshti University of Medical Sciences, Tehran, Iran; ^5^Chronic Respiratory Diseases Research Center, National Research Institute of Tuberculosis and Lung Diseases (NRITLD), Shahid Beheshti University of Medical Sciences, Tehran, Iran

## Abstract

*Purpose*. The histological diagnosis of Mycobacterium tuberculosis (MTB) remains a diagnostic challenge despite different methods. Immunohistochemistry (IHC) not only could confirm granulomatous tissue involvement but also can demonstrate MTB antigen immunolocalization. This study tries to clarify the details of immunohistochemical staining for MTB with pAbBCG. *Materials/Methods*. Twenty-three confirmed TB granulomatous tissue samples were studied by Ziehl-Neelsen and immunohistochemistry (IHC) staining with pAbBCG. Samples were selected from the archive of the Department of Pathology, National Research Institute of Tuberculosis and Lung Disease, Tehran, Iran. *Results*. IHC staining was positive in all samples, whereas Ziehl-Neelsen was positive in 9 cases out of 23 (39.1%). Tissue types used were pleural tissue, lymph nodes, and lung tissue. IHC showed positive coarse granular cytoplasmic and round, fragmented bacillary staining. In this study, epithelioid cells clearly showed more positive staining at the periphery of the granuloma rather than the center of granuloma. There is also positive staining in endothelial cells, fibroblasts, plasma cells, lymphocytes, and macrophages outside the granuloma. *Conclusion*. Considering the criteria of positive immunohistochemical staining of TB granulomatous reactions, this stain not only highlights the presence of mycobacterial antigens for tissue diagnosis, but also could morphologically localize its distribution in different cells.

## 1. Introduction

Histological diagnosis of tuberculosis (TB) has long been an important issue in anatomical pathology. On the other hand, extrapulmonary TB comprises 10–15% of infections with Mtb. Following HIV prevalence increase, extrapulmonary TB is rising as well [1, 2].

Considering the limitations in sensitivity and specificity of Ziehl-Neelsen staining [3], mycobacterial evaluation, mycobacterial culture, and molecular and serological techniques [4–6], histomorphological analysis appears to be the only feasible technique for field diagnosis of TB in some patients [7, 8].

Granulomatous reactions, and in some cases, nongranulomatous reactions such as the presence of foamy macrophages [9] or mycobacterial spindle cell pseudotumour [10] in some types of mycobacterial infections are indicative of TB only if the presence of TB bacilli has been confirmed in the tissue. Recently, it has been stated that PCR results are acceptable only if the presence of TB bacilli can be confirmed in the tissue [11].

Detection of beaded bacilli (which are more commonly observed in the necrotic zone) by Ziehl-Neelsen staining of tissue specimens [12] indicates the association of tissue reaction with mycobacterial infection. However, Ziehl-Neelsen stain has a relatively low sensitivity for detecting Mtb with sensitivity range of zero to 44% for this infection [13]. Furthermore, presence of a minimum of 10^4^ bacilli per each slide is required to reach diagnosis.

Considering the mechanism of Ziehl-Neelsen staining and its relatively low sensitivity and specificity, this staining technique has limited diagnostic value and inadequate ability for pathophysiological assessment of Mtb antigens in the tissue. Searching for mycobacterial antigens using IHC is a well-recognized technique with special application in research projects. This technique is based on the production of a variety of polyclonal and monoclonal antibodies in tissue reactions. Several studies have reported the sensitivity of this technique can be 64 to 100% for the detection of mycobacterial antigen. Limited numbers of studies have discussed in depth positive tissue staining method for Mtb [14]. However, these techniques have only been briefly mentioned in the pathology reference books and textbooks do not provide a standard for morphological assessment with regard to IHC staining of granulomatous reactions.

Recent studies on the variable morphology of TB bacilli emphasize the importance of IHC in searching for different morphological variations of TB antigens in the tissue.

The present study compares the sensitivity and specificity of the two techniques of Ziehl-Neelsen and IHC staining for field diagnosis of TB. It also evaluates the value of accurate description of IHC staining with pAbBCG in tissues with granulomatous reaction due to TB with regard to diagnosis, immunolocalization, and morphology of TB bacilli in this technique.

## 2. Materials and Methods

A total of 50 patients undergoing simultaneous biopsy and tissue culture with positive tissue culture for Mtb during 2005–2009 were selected from the MRC Department Masih Daneshvari Hospital. Using the archives of the Pathology Department of this hospital, which is a referral center for pathological lung lesions, H&E slides of the selected patients were evaluated. Cases with small number of granulomatous lesions or tissue volume were excluded from the study. Eventually, 23 tissue samples of 23 TB patients with adequate tissue and number of granuloma were chosen for different staining techniques. Characteristics and type of samples were retrieved from the pathology reports. Paraffin-embedded blocks were stained using Ziehl-Neelsen and IHC staining. Seven control tissue specimens were also selected from the archives of the Pathology Department. These specimens had granulomatous reactions due to foreign body, fungi, or hydatid cyst. These specimens were also stained similar to the abovementioned samples. Mycobacteriology Research Center of Masih Daneshvari Hospital is the national reference center and educational collaborating center of the WHO office in Eastern Mediterranean Region. The study conducted after Masih Daneshvari Hospital ethic committee.

For immunohistochemical staining, 3-4 micron sections were cut from the tissue block and incubated overnight at room temperature. After deparaffinization and fixation with 99–70% alcohol, specimens were rinsed with distilled water and PBS. In order to remove endogenous peroxidase, methanol and 3% H_2_O_2_ were used for 10 minutes.

After retrieval and cooling down the tissues, they were rinsed with distilled water and PBS and specimens were blocked with antiserum. Antibody was prepared with Tween 20 + Tris (Merck 1.088387) at a 1 : 1000 dilution. In the first phase, primary antibody (DAKO, code no. B 0124) (pAbBCG) was reacted in a wet environment at room temperature for 30 min and after rinsing the specimens with PBS, and secondary antibody (Envision K5007) was added. After staining with chromogen, hematoxylin was used as the contrast dye. For each staining, one negative and one positive control were also considered. Staining was done as fine granular cytoplasmic, coarse granular cytoplasmic and bacillus staining. Different areas and cells in tissue specimens were evaluated; type of granuloma (with or without necrosis), and presence of multi-nuclear giant cells, epithelioid cells, necrotic zones, lymphocytes, plasma cells, peri-granuloma macrophages and fibroblasts and perigranuloma endothelial cells were assessed by a junior and a senior pathologist. Positive staining was defined as staining of 10% of epithelioid cells in the granuloma. For other types of cells, any form of staining was considered as positive.

Ziehl-Neelsen staining was performed according to the standard protocol. In summary, tissue specimens were deparaffinized and rinsed with consecutive dilutions of alcohol (96% to 70% ethanol). After heat fixation, specimens were washed with carbol fuchsin for 4 minutes and incubated with HCL. Counterstaining was done using Brilliant Green for 20 s. After rinsing, samples were allowed to dry at room temperature.

## 3. Results

**Figure 1:**
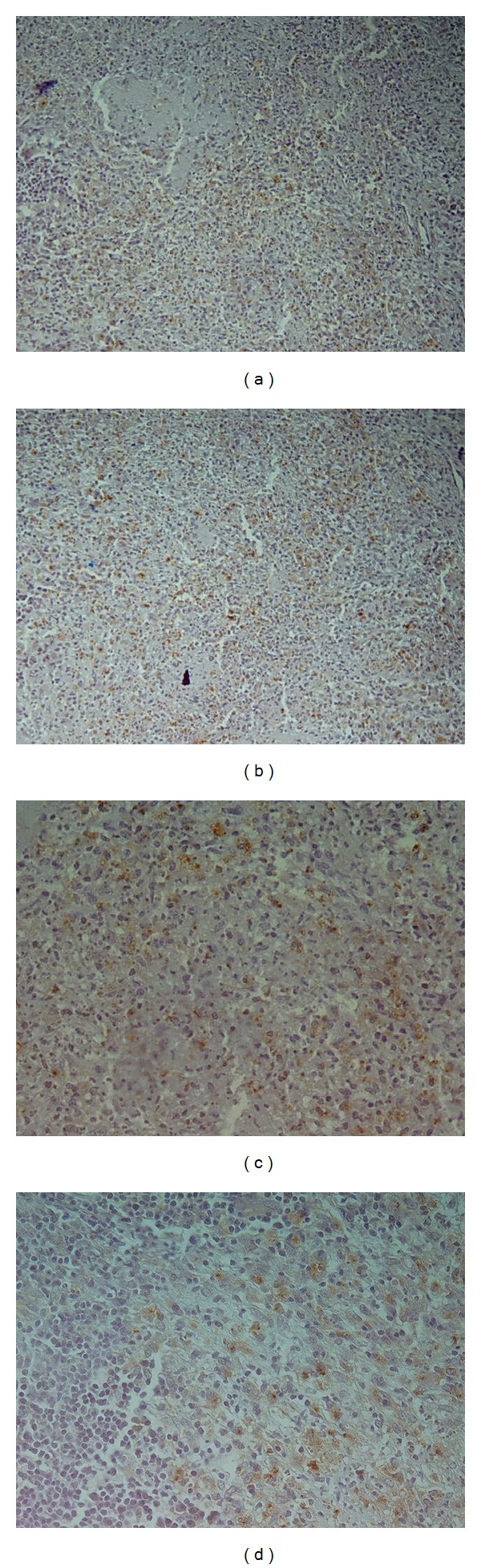
Necrotizing granulomatous reaction with course granular cytoplasmic positioning in epithelioid histiocyte ((a) and (b) ×10, (c) ×20, and (d) ×40).

**Figure 2:**
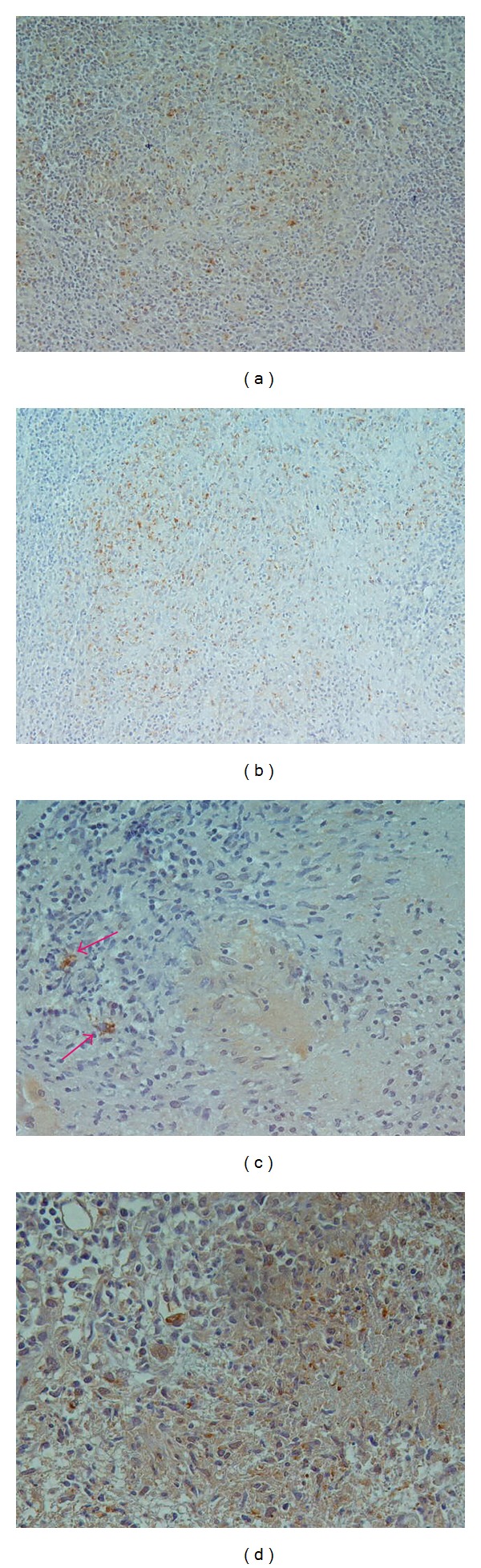
IHC for pAbBCG (×20): course cytoplasmic positivity of epithelioid macrophage without background in epithelioid histiocyte in nongranulomatous reaction in lymph node ((a) and (b)). Course granular cytoplasmic positivity of macrophage outside the granulomatous process ((c) and (d)).

A total of 23 cases were evaluated, out of which 17 (73.9%) were males. Type of tissue in understudy cases was pleura (9 cases, 39.1%), lymph node (cervical, axillary, and thoracic) (9 cases, 39.1%), and lung tissue (5 cases, 21.7%). Granuloma, in the form of necrotizing poorly organized granuloma, was 9 cases (39.1%), necrotizing well-organized granuloma in 5 cases (21.7%), necrotizing nonpoorly organized granuloma in 4 cases (17.3%), and non-necrotizing well-organized granuloma observed in 3 cases (13%). In all biopsy samples, IHC staining yielded positive results for both fine and coarse granular cytoplasmic and round bacillary staining in epithelioid cells (Figures 1 and 2). Staining of other cells was also positive in varying percentages. These shapes were variable from fragmented bacilli to antigen dust. Ovoid and circular shapes were also observed. In granulomatous reactions in the control group (which were all non-TB), IHC staining was only in the form of fine granular cytoplasmic staining in epithelioid cells and focal staining was also seen in some other cells outside the granuloma. Coarse granular cytoplasmic staining, fragmented or round bacillary staining, was not observed in any of the cases. Ziehl-Neelsen staining of all control cases was negative. In test patients, 9 out of 23 (39.1%) had positive Ziehl-Neelsen staining.  Table 1 shows the positive results and comparison of the sensitivity and specificity of pAbBCG and Ziehl-Neelsen.

**Figure 3:**
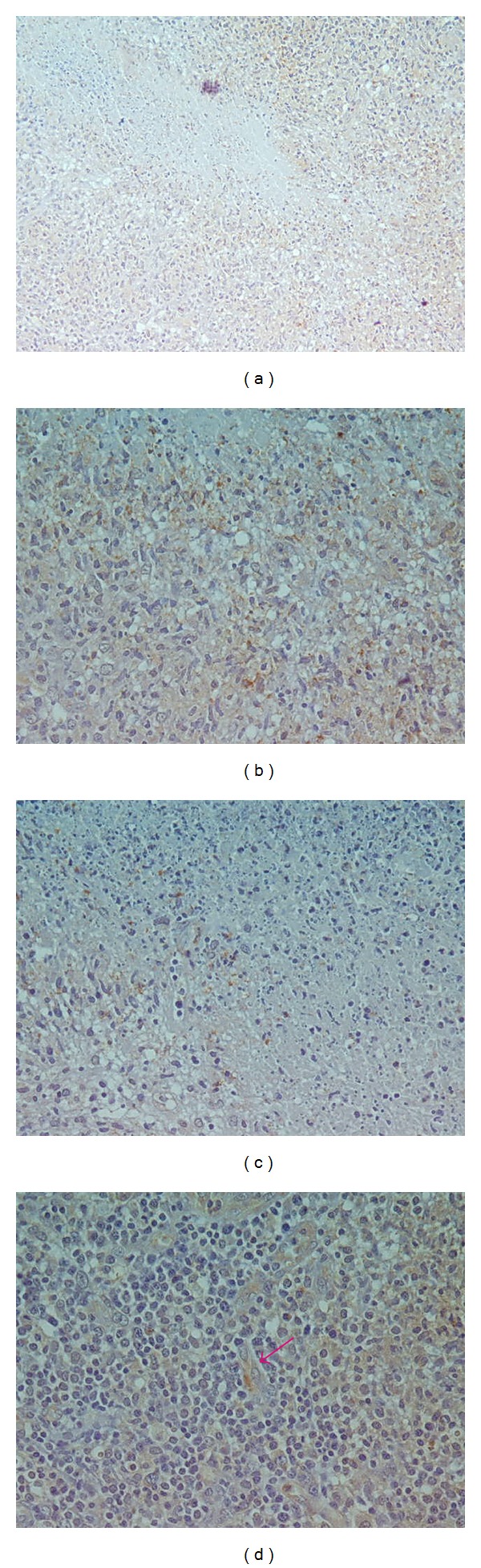
Fine antigen dust cytoplasmic positivity in plasma cell and lymphocyte, area of necrosis in the top without prominent positivity in contrast of coarse granular positivity of histiocyte. ((a) ×10, (b) and (c) ×20). Coarse granular and fine antigen dust in endothelial cells ((d) ×20).

In all 23 cases, coarse granular cytoplasmic staining and stained intact bacilli along with lower degrees of fine granular cytoplasmic staining were observed in epithelioid cells. According to cell types, positive IHC staining was observed in endothelial cells, epithelioid cells, fibroblasts, giant cells, lymphocytes, macrophages, and plasma cells, 2 (8.7%), 19 (82.6%), 3 (13%), 5 (21.7%), 1 (4.3%), 8 (34.8%), and 1 (4.3%), respectively, (Figure 3). Necrotic zone was also positive in IHC staining in 5 cases (21.7%). In this study, epithelioid cells at the periphery of the granuloma were more positively stained than the epithelioid cells at the center or adjacent to the necrotic zone.

## 4. Discussion

Ziehl-Neelsen stain method for the diagnosis of TB is positive in one-third of cases with confirmed TB infection. Detection of tuberculosis in tissue slides is still based on the histological pattern of the granuloma that has several differential diagnoses with different treatments [13].

Presence of mycobacterial antigens and tissue morphology can be evaluated with IHC technique. Eliminating background with proper technique could provide 100% specificity if coarse granular cytoplasmic and bacillus staining are considered. These obtained results are comparable with the findings of a study by Goel and Budhwar, reporting 64–100% sensitivity for IHC and zero to 44% sensitivity for Ziehl-Neelsen staining [14].

Moreover, the results of monoclonal antibody testing [15] also show 100% sensitivity, which is in accordance with our obtained results. This agreement seems to be due to the appropriate staining and accurate consideration of positive staining results.

In order to achieve appropriate staining by this technique, great attention must be paid to dilution and technique of IHC with polyclonal antibodies. If adequate attention is not paid, fine granular staining in the background can cause false positive reactions provided that the pathologist is not familiar with this technique. This is among the main limitations of working with this type of antibody. However, in case of positivity of this staining as coarse granular cytoplasmic staining, stained fragmented bacillia or stained bacilli form, percentage of specificity for this staining technique is highly increased. It should be emphasized that coarse granular cytoplasmic staining or positively stained fragmented bacilli of any degree were not observed in control samples. These findings are in accordance with those of Madhu Goel and Budhwar, describing small bacillary fragments and antigenic dusts [14].

On the other hand, new findings have demonstrated that morphological modifications of tubercle bacilli in circular or intermediate shapes even in the tissue can be live TB bacilli. This issue has been well described by Velayati et al. [16]. In the present study, stained fragments and other shapes indicate morphological alterations of TB bacilli in the tissue.

Another finding is the presence of fragments of TB bacilli in macrophages, fibroblasts, plasma cells, lymphocytes and even endothelial cells outside the granuloma indicating that the mentioned cells play an active role in histopathogenesis of TB.

A similar study [15] mentioned the presence of mycobacterium and intracellular mycobacterium material outside the granuloma and in macrophages and even lymphocytic fragments around the granuloma. Recent studies are also indicative of the active role of endothelial cells in immunologic tissue reactions [17]. Furthermore, the role of plasma cells [18] and fibroblasts [19] alongside the main cells in cell-mediated immune response to TB is a matter of discussion.

Thus, aside from the positivity of lymphocytes for the presence of TB bacilli antigens in IHC staining with pAbBCG, our study is uniquely important in revealing the presence of these antigens in plasma cells, endothelial cells, and fibroblasts. This issue needs to be further investigated in terms of TB pathogenesis.

Ziehl-Neelsen staining has low sensitivity and requires the presence of intact bacilli. Logani et al. [10] demonstrated that IHC staining with pAbBCG yields positive results even in tissues with 10 bacilli per slide. Thus, this technique can be applied even in cases with small number of bacilli for any reason (such as in paucibacillary extrapulmonary TB). This technique can be used in primary stages of TB infection and HIV as a routine pathology test.

Our study showed that the IHC technique has high sensitivity and specificity. It also indicates localization of cells infected with mycobacterium or cells containing TB bacilli antigens. It somehow reveals the dissemination of TB bacilli and its antigens in tissues as well. In this study, epithelioid cells in the periphery of granuloma were stained more positively than the epithelioid cells at the center of granuloma or adjacent to the necrotic zone. Using anti-MPT64 in tissues affected with TB granuloma, Purohit et al. [15] found that epithelioid cells outside the necrotic zone showed greater positivity than cells within the necrotic area. This finding is in contrast to Ziehl-Neelsen staining that mostly reveals intact bacilli at the center of necrotic zone.

There are some limitations to this study. First, we have selected 23 cases from 50 specimens that were culture positive for MTB; thus, the study has bias to show sensitivity and specificity exactly. Secondly, pAbBCG is polyclonal antibody for Mtb complex. Although we have compared the sensitivity and specificity of this method with Ziehl-Neelsen staining, the aim of study is illustration of immunolocalization of Mtb Ag for use in diagnostic or research field, not comparing sensitivity with specificity. Further studies should be carried out on suspected tissues involvement with Mtb bacilli through IHC and use of monoclonal antibodies.

Furthermore, pathologists must be acquainted with adequate staining pattern, elimination of background staining, and type of selected antibody. This method is especially important for use in countries with high prevalence of TB as a technique with early diagnostic value in tissue specimen. In early diagnosis, this technique can reduce related morbidity and mortality and decrease the rate of complications due to misdiagnosis and mistreatment of TB.

## Figures and Tables

**Table 1:** Comparison of the sensitivity and specificity of pAbBCG and Ziel-Neelsen.

Variable	No. (%)	Positive finding	Culture positive	Sensitivity (%)	Specificity (%)	Positive predictive value (%)	Negative predictive value (%)
pAbBCG							
Case	23	23	23	100	100	100	33/3
Control	7	0	0				
Ziel-Neelsen							
Case	23	9	9	39/1	100	100	33/3
Control	7	0	0				
